# *Astragalus membranaceus* Extract Attenuates Inflammation and Oxidative Stress in Intestinal Epithelial Cells via NF-κB Activation and Nrf2 Response

**DOI:** 10.3390/ijms19030800

**Published:** 2018-03-10

**Authors:** Simona Adesso, Rosario Russo, Andrea Quaroni, Giuseppina Autore, Stefania Marzocco

**Affiliations:** 1 Department of Pharmacy, University of Salerno-Via Giovanni Paolo II, 132-84084 Fisciano-Salerno, Italy; sadesso@unisa.it (S.A.); autore@unisa.it (G.A.); 2 Giellepi S.p.A. Health Science Department, Via Benvenuto Cellini 37, 20851 Lissone (Monza Brianza), Italy; rosario.russo@giellepi.it; 3 Department of Biomedical Sciences, Cornell University, Veterinary Research Tower, Cornell University, Ithaca, NY 14853-6401, USA; aq10@cornell.edu

**Keywords:** *Astragalus membranaceus*, intestinal epithelial cells, inflammation, oxidative stress

## Abstract

*Astragalus membranaceus*, dried root extract, also known as *Astragali radix*, is used in traditional Chinese medicine as a tonic remedy. Moreover, it has been reported that *Astragalus membranaceus* could attenuate intestinal inflammation; however, the underlying mechanism for its anti-inflammatory activity in intestinal epithelial cells (IECs) remains unclear. In this study, we evaluated *Astragalus membranaceus* extract (5–100 µg/mL) in a model of inflammation and oxidative stress for IECs. We showed that *Astragalus membranaceus* extract reduced the inflammatory response induced by lipopolysaccharide from *E. coli* (LPS) plus interferon-γ (IFN), decreasing tumor necrosis factor-α (TNF-α) release, cycloxygenase-2 (COX-2) and inducible nitric oxide synthase (iNOS) expression, nitrotyrosine formation, nuclear factor-κB (NF-κB) activation, and reactive oxygen species (ROS) release in the non-tumorigenic intestinal epithelial cell line (IEC-6). The antioxidant potential of *Astragalus membranaceus* extract was also evaluated in a model of hydrogen peroxide (H_2_O_2_)-induced oxidative stress in IEC-6, indicating that this extract reduced ROS release and increased nuclear factor (erythroid-derived 2)-like 2 (Nrf2) activation and the expression of antioxidant cytoprotective factors in these cells. The results contributed to clarify the mechanisms involved in *Astragalus membranaceus* extract-reduced inflammation and highlighted the potential use of this extract as an anti-inflammatory and antioxidant remedy for intestinal diseases.

## 1. Introduction

Inflammatory bowel disease (IBD) is one of the most prevalent gastrointestinal disorders, and it includes ulcerative colitis (UC) [1] and Crohn’s disease (CD) [2]. IBD pathogenesis results from a multifactorial process involving genetic, environmental, and immunogenic factors [3]. Over the last few decades, our understanding of IBD aetiology has increased, yet its exact mechanisms remain unclear. Nevertheless, it is well known that IBD is characterized by inflammation and abnormalities in epithelial barrier function [4]. Current knowledge shows that intestinal epithelial cells (IECs), which under physiological conditions are indispensable to maintain a selective barrier between the host and harmful substances present in the lumen, have emerged as key players in the generation and persistence of intestinal inflammation during IBD [5]. Different noxious agents, such as chemical, physical, infectious, and inflammatory injuries can damage the intestinal epithelial integrity. This damage can lead to increased penetration and absorption of toxic substances, the activation of immunogenic responses, and a final disequilibrium in the host’s homeostasis. In response to these external factors, IECs and immune cells are activated and can trigger an inflammatory response [6]. The release of pro-inflammatory factors, such as cytokines and chemokines, as well as oxidative stress with the release of reactive oxygen species (ROS), are the main events taking place in intestinal inflammation during the active phase. In particular, by influencing transcription factors and redox-sensitive signaling pathways, ROS and their oxidized products can further sustain inflammation within the intestinal layer.

Plants are considered a potential source of antioxidant and anti-inflammatory molecules. The genus *Astragalus* belongs to the legume family (Fabaceae). This genus, the largest of flowering plants in the world, includes 2500–3000 species mainly found in Central and Southwestern Asia [7,8]. Different *Astragalus* species are used in traditional medicine, mostly Chinese, and the dried roots of *A. membranaceus* (Fisch.) Bge and *A. membranaceus* (Fisch.) var. *mongholicus* (Bge) Hsiao are included in the drug *Huangqi* (*Radix Astragali*), which is present in the pharmacopoeia of the People’s Republic of China [9]. *Astragalus membranaceus* is used as a tonic and has many effects, such as enhancing defensive energy and inducing diuresis to treat edema [10]. It is widely used in East Asia to prevent some severe chemotherapy side effects [11] and liver fibrosis [12]. Moreover, recent pharmacological studies and clinical evidence centered on *Astragalus membranaceus* have reported a wide spectrum of biological activities for this plant [13,14], including at an intestinal level [15,16,17]. *Astragali radix*, the dried root of *Astragalus membranaceus*, is a popular health-promoting herb, and its use as a crude drug is one of the oldest and most frequently used remedies in oriental medicine [18]. Pharmacological studies have demonstrated that the water extract of *Astragali radix* possesses many biological functions [19,20,21,22,23]. Also, *Astragalus* polysaccharides, which are major constituents of *Astragali radix*, possess many biological effects and pharmacological properties, including at intestinal levels [24,25]. Despite this, there is little mechanistic knowledge regarding the molecular action(s) of *Astragalus membranaceus* root extract on IECs [26] and especially during inflammatory conditions. Considering the pivotal role of IECs in maintaining and regulating intestinal homeostasis, in this study, we evaluated the effects of *Astragalus membranaceus* extract (5–100 µg/mL) on inflammation and oxidative stress in the intestinal epithelial cell line (IEC-6) in order to elucidate the effect of *Astragalus membranaceus* extract during intestinal inflammation at the cellular level.

## 2. Results

### 2.1. Astragalus membranaceus Extract Did Not Have Any Antiproliferative Activity on IEC-6 Cells

To evaluate the antiproliferative potential of *Astragalus membranaceus* extract on IEC-6, cells were treated with the extract (5–100 μg/mL) for 24 h. The results indicated that the extract did not have any significant antiproliferative activity on IEC-6 cells (mean ± SEM of % antiproliferative activity vs. control: 1.12 ± 1.04, 3.38 ± 1.20, 4.06 ± 1.15, 6.16 ± 2.03, respectively for *Astragalus membranaceus* extract 5, 10, 50, 100 μg/mL).

### 2.2. Astragalus membranaceus Extract Reduced Tumor Necrosis Factor-α (TNF-α) Levels in Lipopolysaccharide from E. coli (LPS) + Interferon-γ (IFN)-Stimulated IEC-6

**Figure 1:**
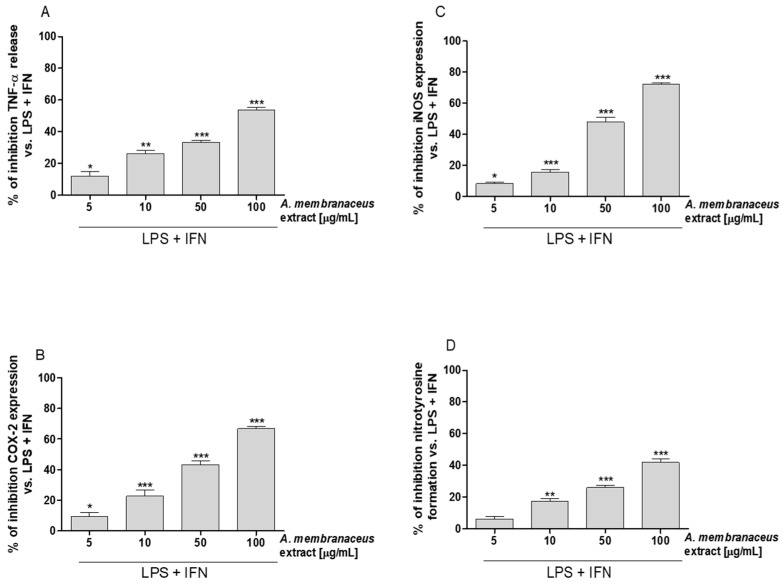
Inhibitory and concentration related effect of *Astragalus membranaceus* extract (5–100 μg/mL) in LPS + IFN-stimulated IEC-6 on (**A**) tumor necrosis factor-α (TNFα) levels, evaluated using an ELISA, (**B**) cyclooxygenase-2 (COX-2) expression, (**C**) inducible nitric oxide synthase (iNOS) expression, and on (**D**) nitrotyrosine formation, evaluated by the cytofluorimetric technique. Data are expressed as mean ± SEM. ***, **, * indicate *p* < 0.001, *p* < 0.01 and *p* < 0.05 vs. LPS + IFN.

The effect of *Astragalus membranaceus* extract on TNF-α levels in IEC-6 cellular medium was evaluated using an enzyme-linked immunosorbent assay (ELISA). Our results showed that *Astragalus membranaceus* extract (5–100 μg/mL) significantly inhibited TNF-α release, induced by LPS + IFN, in IEC-6 cells medium (*p* < 0.05 vs. LPS + IFN; Figure 1A).

### 2.3. Astragalus membranaceus Extract Reduced Cycloxygenase-2 (COX-2) and Inducible Nitric Oxide Synthase (iNOS) Expression and Nitrotyrosine Formation in LPS + IFN-Stimulated IEC-6

Expression of COX-2 and iNOS were evaluated by a cytofluorimetric technique. Our results showed that *Astragalus membranaceus* extract (5–100 μg/mL) inhibited COX-2 and iNOS expression in IEC-6 cells at all tested concentrations (*p* < 0.05 vs. LPS + IFN; Figure 1B,C). Under the same experimental conditions, the extract (5–100 μg/mL) also inhibited nitrotyrosine formation in IEC-6 cells (*p* < 0.01 vs. LPS + IFN; Figure 1D).

### 2.4. Astragalus membranaceus Extract Reduced p65 Nuclear Factor-κB (NF-κB) Translocation in LPS + IFN-Stimulated IEC-6

**Figure 2:**
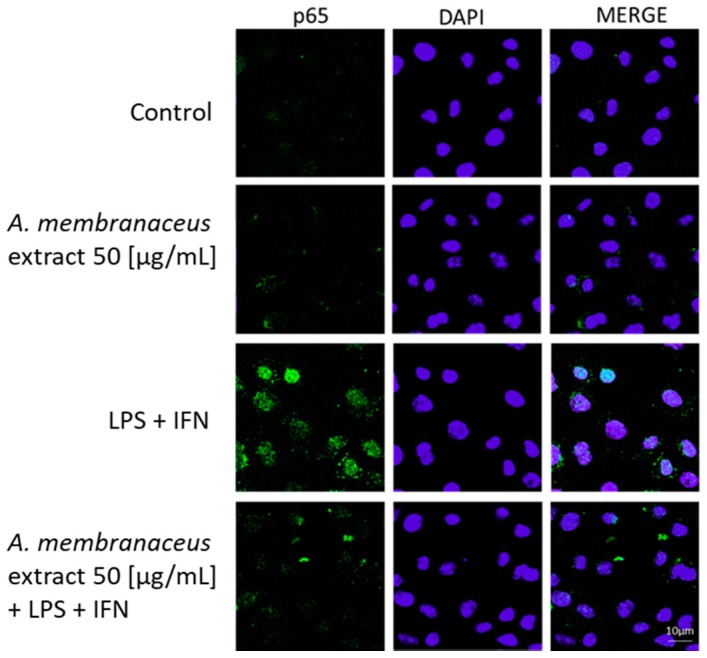
Effects of *Astragalus membranaceus* extract (50 μg/mL) alone and with LPS + IFN on nuclear factor-κB (NF-κB) p65 nuclear translocation, evaluated by immunofluorescence analysis. The blue fluorescence identified the nuclei, while the green fluorescence indicated the p65 NF-κB subunit.

To evaluate NF-κB activation, p65 NF-κB was labeled with a green fluorescent marker. *Astragalus membranaceus* extract alone did not induce p65 nuclear translocation in IEC-6 cells (Figure 2). However, at a concentration of 50 μg/mL, the extract inhibited p65 NF-κB nuclear translocation when compared to LPS + IFN treatment alone (Figure 2).

### 2.5. Astragalus membranaceus Extract Reduced ROS Release by IEC-6 Cells

**Figure 3:**
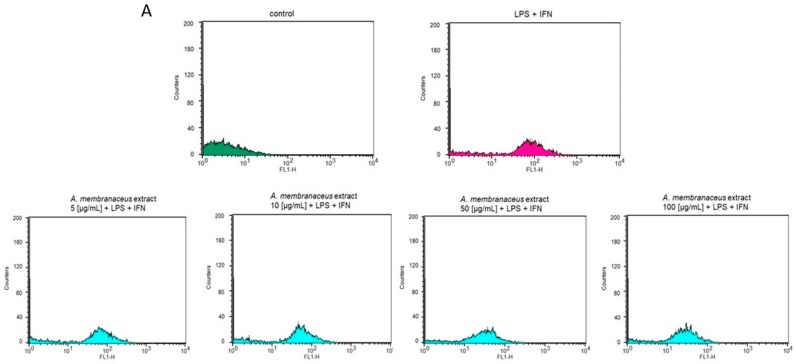

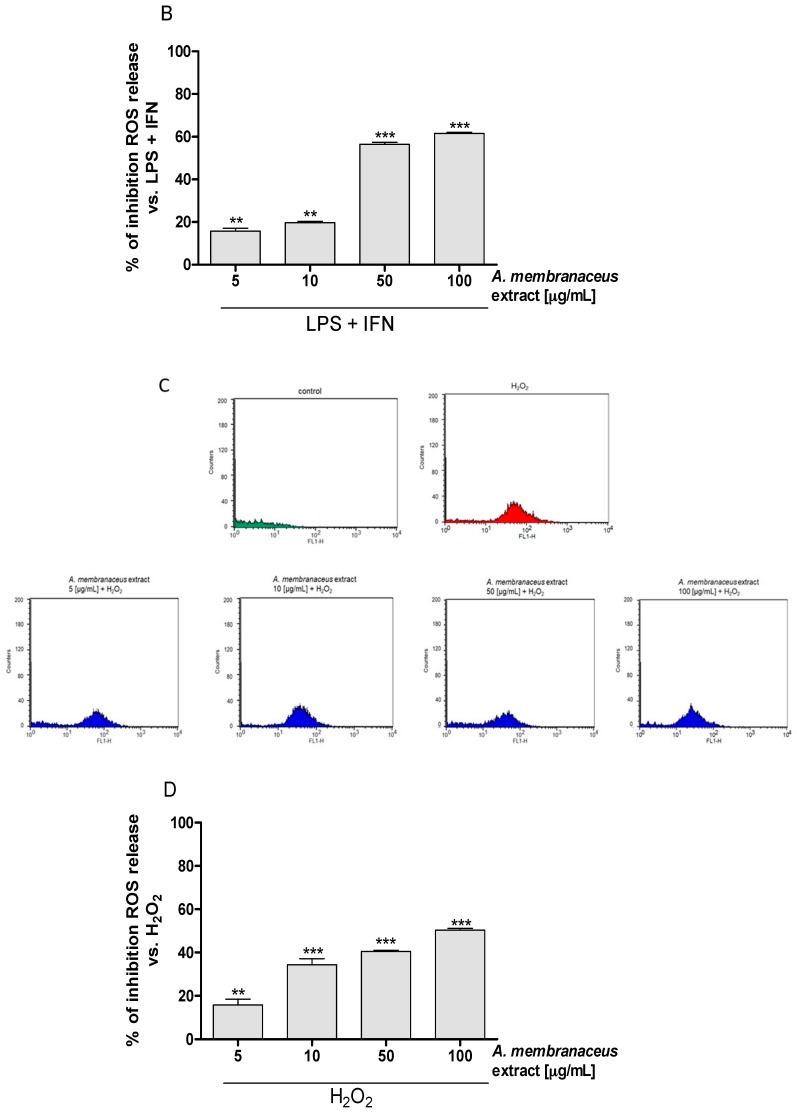
(**A**,**B**) Effect of a graded concentration of *Astragalus membranaceus* extract (5–100 μg/mL) in the presence of LPS + IFN on reactive oxygen species (ROS) levels, detected by 2′,7′ dichlorofluorescein-diacetate (H_2_DCF-DA). (**C**,**D**) Effect of *Astragalus membranaceus* extract (50 μg/mL) in the presence of H_2_O_2_ on ROS formation, evaluated with the probe H_2_DCF-DA. Data are expressed as mean ± SEM. ***, ** denote *p* < 0.001 and *p* < 0.01 vs. LPS + IFN or vs. H_2_O_2_.

The antioxidant potential of *Astragalus membranaceus* extract was evaluated by measuring the intracellular ROS production in LPS + IFN-stimulated IEC-6 cells. It was found that the root extract (5–100 μg/mL) significantly inhibited ROS production in IEC-6 cells (*p* < 0.01 vs. LPS + IFN; Figure 3A,B). To further evaluate its antioxidant potential, *Astragalus membranaceus* extract (5–100 μg/mL) was also evaluated in IEC-6 cells treated with the pro-oxidant stimulus H_2_O_2_ (1 mM). Again, under these different experimental conditions, as assessed during inflammatory conditions, *Astragalus membranaceus* exhibited significant antioxidant activity by inhibiting ROS release (*p* < 0.01 vs. H_2_O_2_; Figure 3C,D).

### 2.6. *Astragalus membranaceus* Extract Induced Nuclear Factor (Erythroid-Derived 2)-Like 2 (Nrf2) Activation and Heme Oxygenase 1 (HO-1), NAD(P)H Quinone Dehydrogenase 1 (NQO1) Expression in IEC-6 Cells

**Figure 4:**
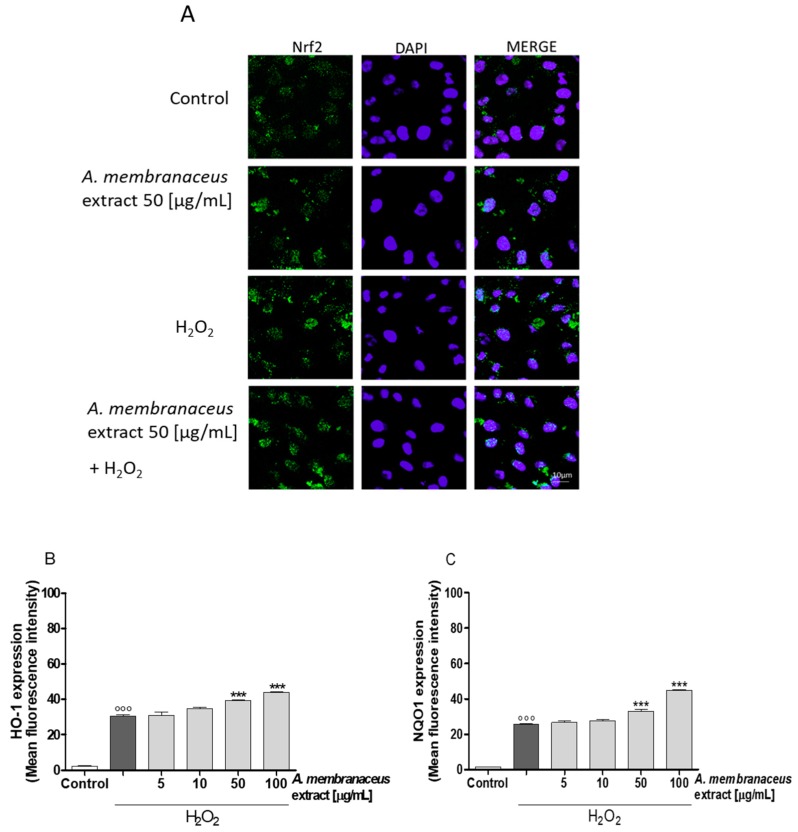
(**A**) Effect of *Astragalus membranaceus* extract (50 μg/mL) alone and in the presence of H_2_O_2_ on the translocation of nuclear factor (erythroid-derived 2)-like 2 (Nrf2) into the cellular nuclei, evaluated by immunofluorescence analysis. The blue fluorescence identified the nuclei while the green fluorescence indicated Nrf2. (**B**) Effect of *Astragalus membranaceus* extract (5–100 μg/mL) in the presence of H_2_O_2_ on heme oxygenase 1 (HO-1) expression, evaluated by the cytofluorimetric technique (**C**) and on NAD(P)H quinone dehydrogenase 1 (NQO1) in IEC-6 cells, evaluated by the cytofluorimetric technique. The dark grey bars (B, C) represent IEC-6 cells treated with H_2_O_2_ alone. Data are expressed as mean ± SEM.°°° denotes *p* < 0.001 vs. control. *** denotes *p* < 0.001 vs. H_2_O_2_.

Oxidative stress is due to a disequilibrium between pro-oxidant and antioxidant factors. To assess the antioxidant potential of *Astragalus membranaceus*, the antioxidant Nrf2 response was also evaluated. As shown in Figure 4A, Nrf2 nuclear translocation in IEC-6 cells was slightly increased one hour after the addition of *Astragalus membranaceus* extract (50 μg/mL) when compared to the untreated control cells. Moreover, Nrf2 activation was increased even more with the addition of the extract under pro-oxidant conditions when compared to cells treated by H_2_O_2_ alone (Figure 4A). Expression of Nrf2-related cytoprotective factors, such as HO-1 and NQO1, was significantly increased in the presence of H_2_O_2_ (*p* < 0.001 vs. control; Figure 4B,C), and it was further increased by *Astragalus membranaceus* extract (*p* < 0.001 vs. H_2_O_2_; Figure 4B,C), thus supporting the antioxidant potential of the latter.

## 3. Discussion

*Astragalus membranaceus*, also known as *Astragali radix*, is a popular health-promoting herb that has been used by people in China to strengthen immunity for more than 2000 years. The effect of *Astragalus membranaceus* has been investigated in models of intestinal injury. In particular, *Astragalus membranaceus* has been reported to have a protective effect during intestinal mucosa reperfusion injury [16]. It has also been reported that *Astragalus membranaceus* has protective effects on small intestine villi and that it increases the level of antioxidant factors, such as superoxide dismutase activity (SOD) [17]. 

While the potential activity of *Astragalus membranaceus* extract as an anti-inflammatory and antioxidant agent has been widely demonstrated in in vivo studies, its effects on IECs, which have a pivotal role in maintaining intestinal homeostasis, are not well understood. For this reason, the aim of this study was to clarify the contribution of *Astragalus membranaceus* extract in the inflammatory process in the intestinal cells and to highlight its potential use as a new anti-inflammatory and antioxidant natural remedy for IBD treatment.

This study provided evidence that *Astragalus membranaceus* extract decreased inflammatory response in IECs by reducing the following: (1) TNF-α production; (2) COX-2 and iNOS expression; (3) nitrotyrosine formation; and (4) NF-κB activation. Interestingly, the extract also exerted an appreciable antioxidant effect by reducing ROS release and by activating antioxidant elements, such as Nrf2 activation and HO-1 and NQO1 expression. 

During the inflammatory process, several pro-inflammatory cytokines were produced by IEC-6 cells, among them TNF-α. TNF-α has a pivotal role in intestinal inflammation, such as in IBD, and clinical results using anti-TNF-α drugs support its role in IBD pathogenesis. TNF-α promotes the activation and recruitment of immune cells and induces apoptosis of IECs and abnormal expression levels of tight junction molecules in these cells factors that ultimately lead to disruption of the intestinal mucosal barrier and increased permeability [27,28,29]. In our experiments, *Astragalus membranaceus* extract significantly reduced TNF-α levels in IEC-6 cells during inflammation. These data are in accordance with previous findings indicating the protective effect of the root extract of *Astragalus membranaceus* in a model of hapten-induced colitis mediated by TNF-α modulation [30] and with the fact that its administration could regulate TNF-α in UC patients [31]. Cytokines and TNF-α lead to the activation and release of other inflammatory mediators, such as iNOS and COX-2, amplifying and perpetuating the inflammatory condition in IBD. COX-2 and iNOS are expressed mainly at the inflammation sites, affecting colon integrity and contributing to the progress of intestinal damage [32], potentially leading to carcinogenesis associated with IBD. In fact, it was also shown that both COX-2 and iNOS stimulated tumor angiogenesis in colorectal cancer, a process mainly induced by vascular endothelial growth factor [33]. Our results showed that COX-2 expression was significantly reduced by *Astragalus membranaceus* extract in LPS + IFN-stimulated IEC-6 at all tested concentrations. The inhibition of COX-2 expression by *Astragalus membranaceus* extract indicated that the extract positively modulated the arachidonic acid cascade during inflammation, thus significantly contributing to its anti-inflammatory effects in IECs. Our findings agree with other studies reporting that *Astragalus membranaceus* reduced COX-2 expression and inflammatory response in general [19,34]. iNOS is the inducible isoform of the nitric oxide synthase, responsible for the synthesis of pro-inflammatory nitric oxide (NO), mainly induced in inflammatory cells, such as macrophages and mononuclear cells neutrophils by pro-inflammatory stimulants [35]. It has been reported that NO levels and iNOS activity increased in the colonic mucosa of UC and CD patients [36]. NO has a pivotal role in many organs and tissues and can exert beneficial or harmful effects depending on its levels and on the redox environment of the site. Its role as a modulator of key signaling pathways and as a regulator of the physiological functions of the gut, such as gastrointestinal motility, absorption, and secretion, is exerted at low NO concentration. The expression of iNOS results in high levels of NO and for a longer time. Therefore, under these conditions, NO regulates many pathophysiologic processes. It is also known that NO produced by the inducible isoform iNOS generates free radicals such as peroxynitrite and hydroxyl radical. Peroxynitrite affects protein function through its ability to nitrate tyrosine residues, thus inducing nitrotyrosine formation. Since tyrosine nitration represents an alternative pathway to phosphorylation of key residues, this process can affect the enzymatic activity of proteins and thus significantly affect intracellular processes [37]. Our results provided evidence for the ability of *Astragalus membranaceus* extract to inhibit iNOS protein expression at all tested concentrations, and this inhibitory effect could lead to significant reduction in NO overproduction and thus to nitrotyrosine formation in LPS + IFN-treated IEC-6 cells. These data are in accordance with previous studies reporting that aqueous extracts of *Astragalus membranaceus* could modulate pro-inflammatory cytokine gene expression and nitric oxide production in macrophages [38]. A nuclear pro-inflammatory activated factor common to all these mediators is NF-κB. NF-κB is a key regulator of inflammation and can be activated by a broad panel of stimuli, including bacterial components such as LPS, proinflammatory cytokines such as TNF-α and interleukin-1, viruses, and DNA-damaging agents [39]. Increased expression and activation of NF-κB were observed in IBD patients, especially in mucosal macrophages and epithelial cells, accompanied by enhanced production of proinflammatory cytokines such as TNF-α, IL-1, and IL-6 [40]. The NF-κB pathway functions as a molecular link between inflammation and tumorigenesis due to its ability to stimulate the expression of proinflammatory cytokines, antiapoptotic factors, angiogenesis factors, and proteases, which promote tumor initiation and ensure the survival and proliferation as well as invasion of malignant cells. Here, we reported that *Astragalus membranaceus* extract can inhibit NF-κB activation, in accordance with previous studies that demonstrated the protective effect of *Astragalus* spp. polysaccharide in inhibiting NF-κB signaling in a colitis animal model [41]. The roles of oxidative stress and the oxidant/antioxidant balance in IBD development have recently received increasing attention. Oxidative stress has been demonstrated to influence different gastrointestinal diseases, such as IBD, gastroduodenal ulcers, and malignancies [42]. In our experimental model, *Astragalus membranaceus* extract exerted a significant antioxidant effect by inhibiting ROS release induced both by LPS + IFN and by H_2_O_2_. These results agree with previous data reporting the antioxidant potential of *Astragalus membranaceus* at the intestinal level [43,44]. Oxidative stress is due to an imbalance between pro- and antioxidant factors; thus, cellular antioxidant response plays a pivotal role in controlling oxidative stress. Nrf2 is a redox-sensitive transcription factor that plays a key role in the antioxidant response. When cells were exposed to oxidative stress, Nrf2 was released from Kelchlike ECH-associated protein 1 (Keap1), which sequesters Nrf2 in the cytoplasm and then binds to Maf or Jun to form a heterodimer in the nucleus. The heterodimer combines with the antioxidant response element (ARE) to promote the expression of genes encoding many phase II detoxification and antioxidant enzymes, including HO-1 and NOQ1, thereby improving the ability of the cell to remove ROS [45]. A Nrf2 deficiency has been shown to exacerbate colonic injury in a mouse model of experimental colitis [46,47], whereas the pharmacological activation of Nrf2 produced protective effects on the colon [48,49]. HO-1 and NQO1 enzymes also exert a protective effect during intestinal inflammation [50]. In the presence of H_2_O_2_, *Astragalus membranaceus* extract enhanced Nrf2 activation and HO-1 and NQO1 expression, thus increasing an antioxidant response in IEC-6 cells. This evidence fits with other data reporting that the *Astragalus* constituents activate Nrf2 cellular response [51]. Several bioactive compounds have been highlighted in the dried root of *Astragalus*, such as polysaccharides, triterpene saponins, isoflavonoids, and various trace elements [52,53]. The phytochemical characterization of the present extracts reveals the presence of these active compounds [52,53]. Studies suggested that *Astragalus* polysaccharides have effects on the activation of B cells and macrophages, promotion of humoral and immune responses, protection of blood vessels, and prevention of inflammation and cancer [54,55]. Moreover, *Astragalus* polysaccharides also have effects at the intestinal level; in particular, they attenuate murine colitis through inflammasome inhibition [25]. The hepatoprotective properties have been widely studied, and it has been found that the *Astragalus* polysaccharides’ antioxidant activity can prevent liver and intestinal damage [13,56,57]. Similarly, the anti-inflammatory effect of triterpene derivatives, also derived from *Astagalus*, has been reported [58,59] and the antioxidant potential of *Astragalus* constituents has been reported and reviewed [60,61,62]. However, despite the activity of *Astragalus* constituents, it is important to underscore that the efficacy of 50% HA (Axtragyl^®^) is strongly related to the whole phytocomplex [53].

In conclusion, our results indicated that the *Astragalus membranaceus* extract exerted significant anti-inflammatory and antioxidant effects in IECs, thus highlighting its potential application in intestinal inflammatory conditions.

## 4. Materials and Methods

### 4.1. Reagents

Unless otherwise specified, all reagents and compounds were purchased from Sigma Chemicals Company (Sigma, Milan, Italy). 

### 4.2. Plant Material

*Astragalus membranaceus* dried root hydroalcoholic extract (Axtragyl^®^) was provided by Giellepi Health Science Division (Lissone, Italy). The extract (50% HA) has already been characterized by Di Cesare Mannelli et al. [52,53] and contains 70% polysaccharides and other bioactive molecules (i.e., saponins and isoflavonoids).

### 4.3. Cell Culture

IEC-6 cells (CRL-1592) were purchased from the American Type Culture Collection (ATCC, Rockville, MD, USA). These cells, derived from normal rat intestinal crypt cells [63], were grown in Dulbecco’s modified Eagle’s medium (DMEM, 4 g/L glucose) with 10% (*v*/*v*) foetal bovine serum (FBS), 2 mM l-glutamine, 1.5 g/L NaHCO3, and 0.1 unit/mL bovine insulin. Cells between the 17th and 21st passages were used for these experiments, as previously reported [64].

### 4.4. Cell Treatment

IEC-6 cells were plated and, after adhesion, were treated with *Astragalus membranaceus* extract (5–100 μg/mL) for 1 h and then co-exposed to *Astragalus membranaceus* extract and lipopolysaccharides from *E. coli* (LPS; 10 μg/mL) plus interferon-γ (IFN; 10 U/mL) for different times, as outlined below. In order to estimate specifically the antioxidant potential of *Astragalus membranaceus* extract, in other experiments, the IEC-6 cells were incubated with *Astragalus membranaceus* extract (5–100 μg/mL) alone for 1 h and then co-exposed to the extract and hydrogen peroxide (H_2_O_2_; 1 mM).

### 4.5. Antiproliferative Activity

IEC-6 cells (5.0 × 10^3^cells/well) were plated on 96-well plates and allowed to adhere for 72 h at 37 °C in a 5% CO_2_ atmosphere. Thereafter, the medium was substituted with either a new one alone or one containing serial dilutions of *Astragalus membranaceus* extract (5–100 μg/mL) and incubated for 24 h. Cell antiproliferative activity was evaluated using the colorimetric assay of 3-(4,5-dimethylthiazol-2-yl)-2,5-diphenyltetrazolium bromide (MTT), as formerly reported [64]. MTT (5 mg/mL) was then added to IEC-6 cells. After 3 h, cells were lysed with 100 µL of a solution containing 50% (*v*/*v*) *N*,*N*-dimethylformamide, and 20% (*w*/*v*) sodium dodecyl sulphate(SDS) (pH = 4.5). A microplate spectrophotometer reader (Titertek Multiskan MCC/340-DASIT, Cornaredo, Milan, Italy) was used to measure the optical density (OD) of released formazan in each well, as we previously reported [65]. The antiproliferative activity was measured as % dead cells = 100 × [(OD treated/OD control) × 100].

### 4.6. TNF-α Determination

TNF-α release in IEC-6 cells was measured with an Enzyme-Linked Immuno Sorbent Assay (ELISA). IEC-6 cells (8.0 × 10^4^ cells/well/24-well plates) were treated as previously reported for 24 h. Supernatants from IEC-6 cells were then collected and a commercial kit (e-Biocscience, San Diego, CA, USA) was used to perform the ELISA, according to the manufacturer’s instructions (e-Biosciences, San Diego, CA, USA). The results were expressed as pg/mL, as formerly reported [66].

### 4.7. Measurement of COX-2, iNOS, HO-1, NQO1 Expression and Nitrotyrosine Formation by Cytofluorimetry

IEC-6 cells were plated into 96-well plates (1 × 10^4^ cells/well) and treated as previously indicated for 24 h in order to evaluate COX-2 and iNOS expression and nitrotyrosine formation in IEC-6 cells. In another set of experiments, the IEC-6 cells were incubated with the extract and H_2_O_2_ as previously indicated, in order to measure HO-1 and NQO1 expression. For this analysis, IEC-6 cells were then collected and washed with phosphate buffered saline (PBS). Fixing solution was added to cells for 20 min and then IEC-6 cells were incubated in fix perm solution for a further 30 min. Anti-COX-2 (BD Transduction Laboratories, Milan, Italy), anti-iNOS (BD Transduction Laboratories, Milan, Italy), anti-nitrotyrosine (Merck Millipore, Milan, Italy), anti-HO-1 (Santa Cruz Biotechnologies, Dallas, TX, USA), or anti-NQO1 (Santa Cruz Biotechnologies, Dallas, TX, USA) antibodies were then added for 1 h. The secondary antibody, in fixing solution, was added to IEC-6 cells and cell fluorescence was then evaluated by a fluorescence-activated cell sorter (FACSscan; Becton Dickinson, Milan, Italy) and analyzed by Cell Quest software (version 4; Becton Dickinson, Milan, Italy), as formerly reported [67].

### 4.8. Immunofluorescence Analysis for NF-κB and Nrf2 by Confocal Microscopy

IEC-6 cells were plated on coverslips in a 12-well plate (2 × 10^5^ cells/well) and allowed to adhere for 24 h. After the adhesion time, the cellular medium was substituted with a new one, either alone or in the presence of *Astragalus membranaceus* extract (50 μg/mL), and treated for 1 h. It was then co-exposed to the extract and LPS (10 μg/mL) plus IFN (10 U/mL) for 1 h in order to evaluate NF-κB activation in IEC-6 cells. In another set of experiments, the IEC-6 cells were incubated with *Astragalus membranaceus* extract (50 μg/mL) alone for 1 h and then exposed simultaneously to *Astragalus membranaceus* extract and H_2_O_2_ (1 mM) for 1 h more in order to measure Nrf2 activation. Cells were then fixed with 4% paraformaldehyde in PBS and permeabilized with 0.1% saponin in PBS. The blocking was performed with bovine serum albumin (BSA) and PBS. Cells were then incubated with rabbit anti-phospho p65 NF-κB antibody (Santa Cruz Biotechnologies, Dallas, TX, USA) or rabbit anti-Nrf2 antibody (Santa Cruz Biotechnologies, Dallas, TX, USA) for 1 h at 37 °C. Then, PBS was used to wash the slides. Next, we added the fluorescein-conjugated (FITC) secondary antibody for 1 h. 4′,6-diamidine-2′-phenylindole dihydrochloride (DAPI) was used for the nuclei, as formerly reported [68]. At the end, we mounted the coverslips in mounting medium and images were taken using the laser confocal microscope (Leica TCS SP5, Leica, Wetzalar, Germany), as formerly reported [68].

### 4.9. Intracellular ROS Release Measurement

ROS intracellular production was evaluated by the probe 2′,7′-dichlorofluorescein-diacetate (H_2_DCF-DA) [69]. The IEC-6 cells were seeded in 24-well plates (8 × 10^4^ cells/well) and allowed to adhere for one day. After adhesion, we incubated cells with *Astragalus membranaceus* extract (5–100 μg/mL) alone for 1 h and then co-exposed to the extract and LPS (10 μg/mL) plus IFN (10 U/mL) for 24 h. In another set of experiments, the IEC-6 cells were treated with *Astragalus membranaceus* extract (5–100 μg/mL) alone for 1 h and then exposed simultaneously to the extract and H_2_O_2_ (1 mM) for 1 h more. After cellular treatment, IEC-6 cells were collected, washed with PBS, and then incubated in PBS containing H_2_DCF-DA (10 μM). Cell fluorescence was evaluated after 15 min at 37 °C, using a fluorescence-activated cell sorter (FACSscan; Becton Dickinson, Franklin Lakes, NJ, USA), and was analyzed by Cell Quest software version 4 (Becton Dickinson, Milan, Italy), as formerly reported [69].

### 4.10. Data Analysis

We reported data as mean ± standard error mean (SEM) of at least three independent experiments. Each experiment was conducted in triplicate. For the statistical analysis, we used the analysis of variance test. Bonferroni’s test was used to make multiple comparisons. We considered significant a *p*-value less than 0.05.

## 5. Conclusions

Our results indicated that *Astragalus membranaceus* root extract significantly reduced the inflammatory response and the pro-oxidant status in IEC-6 cells. Our study provides evidence that might further the development of *Astragalus membranaceus* root extract as a therapeutic agent for IBD treatments.
